# Physician and pharmacist satisfaction and clinical needs for the real-time medication surveillance program in South Korea

**DOI:** 10.1186/s12913-019-4686-9

**Published:** 2019-11-06

**Authors:** Sun Mi Shin, Hong-Ah Kim, Inmyung Song, Ha-Lim Jeon, Ju-Young Shin

**Affiliations:** 1 Korea Institute of Drug Safety and Risk Management, Anyang-si, Gyeonggi-do South Korea; 2 School of Pharmacy, Sungkyunkwan University, 2066, Seobu-ro, Jangan-gu, Suwon, Gyeonggi-do 16419 South Korea; 3 College of Nursing and Health, Kongju National University, Gongju, South Korea

**Keywords:** Medication surveillance, Healthcare providers, Satisfaction, Clinical needs, Survey

## Abstract

**Background:**

Since December 2010, a nationwide real-time medication surveillance program has been implemented in Korea to prevent potential adverse drug reactions. Our goal was to evaluate physicians’ and pharmacists’ satisfaction and clinical needs for the medication surveillance program in Korea.

**Methods:**

Both web- and paper-based surveys were conducted using a structured questionnaire among 1164 physicians and pharmacists from May 23, 2014 to August 11, 2014. The survey consisted of questions about the participant’s satisfaction with the medication surveillance program, clinical usefulness, clinical need for the medication surveillance program, and sociodemographic characteristics. Multivariate ordinal logistic regression was performed to investigate the factors influencing satisfaction levels with the medication surveillance program.

**Results:**

We analyzed data from 1120 respondents, including 503 physicians and 617 pharmacists. Overall, 63.1% of the respondents were satisfied with the medication surveillance program. Pharmacists were more satisfied with the program than were physicians (69.1% vs. 55.6%; adjusted odds ratio, 2.13; 95% confidence interval, 1.65–2.76). Among the respondents, 77.8% cited a decrease in therapeutic duplication to be a major improvement resulting from the medication surveillance program, 82.6% considered the drug–drug interaction information useful, and 48.7% suggested that the program should include information on liver or kidney disease–drug interaction.

**Conclusions:**

Overall, 63.0% of physicians and pharmacists were satisfied, and a decrease in therapeutic duplication was regarded as the most beneficial component. Further improvements by considering clinical needs of physicians and pharmacists will be needed to increase satisfaction.

## Background

Inappropriate drug use can cause adverse drug reactions (ADRs), influence public health, and increase medical expenditures because of unnecessary hospital admissions [1]. For use as a tool to prevent inappropriate drug use, the medication surveillance program (so-called Drug Utilization Review) is a structured, concerted effort to evaluate prescription drug use according to pre-determined standards [2]. Its purpose is to continuously improve the quality of pharmacotherapy by evaluating the appropriateness of prescribed drugs, dosages, administration periods, and quality-of-life attributable to medications [3]. Although details are different from country to country, the program usually addresses issues, such as drug–drug interactions, drug–disease interactions, drug–patient precautions, and drug dosage modifications [4]. The medication surveillance program can ensure the standard quality of pharmacotherapy and safe drug use by reducing the risk of abuse, under- and over-use of medications, and preventing ADRs. Furthermore, the medication surveillance program can reduce variation in patient care among prescribers and regions by standardizing drug therapy and reduce medical care costs attributable to unnecessary drug use [5].

**Fig. 1:**
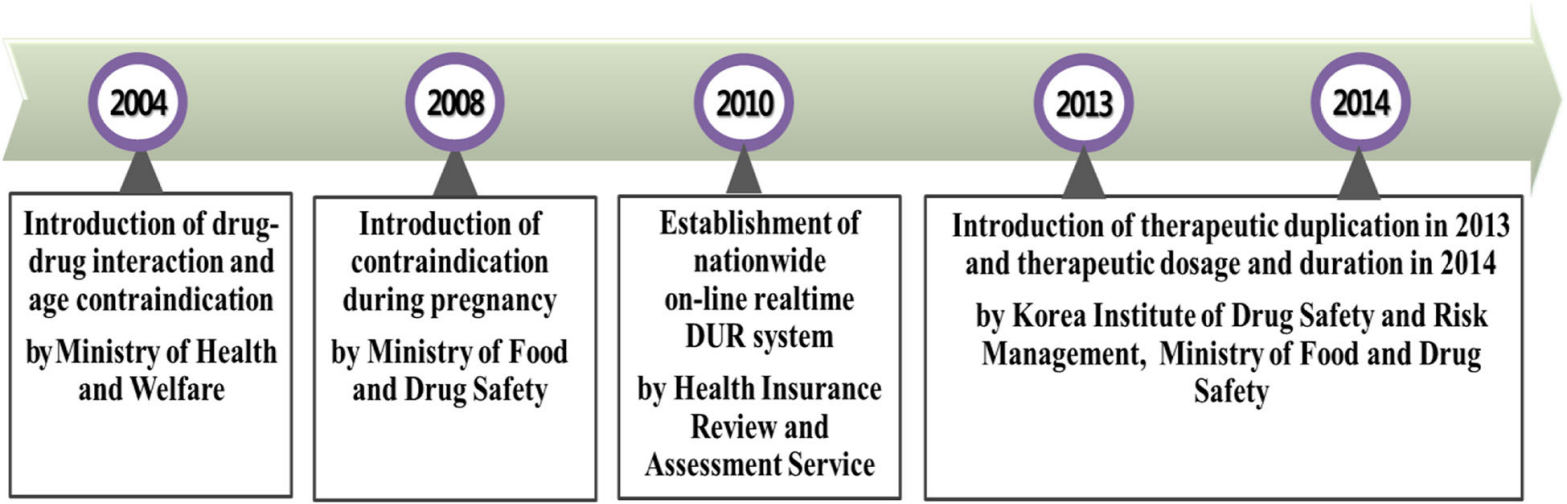
History of the National Medication Surveillance Program in Korea (Source: Personal collection)

The development of a national drug database for medication surveillance began in earnest when the Ministry of Health and Welfare of Korea announced a list of drug–drug interactions and age contraindication drugs in 2004 [6]. However, since 2005, the Korea Ministry of Food and Drug Safety (MFDS) has been in charge developing and announcing the drug lists. In December 2010, the computerized real-time medication surveillance system was established and operated by the Health Insurance Review and Assessment Service (HIRA). The Korea Institute of Drug Safety and Risk Management (KIDS), launched in April 2012, took the role of actively developing a drug database for the medication surveillance program. Since then, the type of surveillance has become more diverse by including not only drug–drug interaction, age and pregnancy contraindications but also therapeutic duplication, dosage advisements, and administration period advisements to prevent duplicative use and overuse of medications and as a result cast a wider and more secure net for drug safety (Fig. 1).

In Korea, when a physician writes a prescription, the information is transmitted to the HIRA and checked in real time through a concurrent system. The prescription is compared to the patient’s medication history and a list of suspended drugs. If it is determined to have any safety issue, the prescriber receives a warning on the computer screen within 0.5 s. Once having received a message, the prescriber can change the prescription or proceed with the original prescription by entering the reason for the override. The pharmacist that dispenses the prescribed medication undertakes the same process and double checks the prescription. If given an alert, the pharmacist can choose to change the prescription or proceed to dispense the prescribed medication. According to the report published by the HIRA in 2018, the medical surveillance system was established in 99.7% of all medical institutions and pharmacies and used by 97.7% of these [7].

The US and European countries that initiated medication surveillance programs earlier experienced alert fatigue, which refers to the phenomenon of habitually overriding pop-up alerts because of frequent occurrences of clinically insignificant, minor alerts [8–11]. This situation is concerning as it can defeat the purpose of the surveillance that was established for patient safety. Diagnosing this problem necessitates evaluating the level of satisfaction with the medication surveillance among healthcare professionals and understanding the inconveniences experienced because of this surveillance program. The scope of the medication surveillance program provided in Korea is narrower than that in other countries because of a lack of information on drug allergies and renal disease precautions [8, 9]. To maximize the usefulness of the medication surveillance program, we need to understand what more is needed by the clinical practice, as well as to prioritize areas of the greatest need. Therefore, the goals of this study were to evaluate physicians’ and pharmacists’ satisfaction levels with the medication surveillance program, because they are the main users of the surveillance services, and to investigate the areas where improvement is needed. In addition, because physicians and pharmacists use somewhat different user interfaces and see patients under different circumstances, we hypothesized that they will have different responses regarding their prescription checking processes and the surveillance service performance. Hence, we compared satisfaction and clinical needs with respect to the program between physicians and pharmacists.

## Methods

### Questionnaire development

We developed a structured questionnaire to investigate the level of satisfaction with the medication surveillance program and to identify the areas of additional need for healthcare professionals. An initial draft of the questionnaire was pilot tested independently with 2 physicians and 5 pharmacists. After the test, we slightly modified the wording of some questions to improve the understanding of the respondents. The questions regarding the characteristics of study participants included profession (physician vs. pharmacist), gender, age, region, and years of practice. The level of satisfaction was collected using a 5-point scale: strongly satisfied, satisfied, neutral, dissatisfied, and strongly dissatisfied. Respondents were asked to rate, using the same scale, their opinions on whether the medication surveillance had made the following specific improvements: prevention of adverse drug reaction, decrease in therapeutic duplication, and decrease of drug overuse. The respondents then rated the degree to which the medication surveillance program was useful in their clinical practice for each information type (drug–drug interaction, age contraindication, pregnancy contraindication, and therapeutic duplication) on the same scale. The respondents were also asked to select the areas that most needed additional information among the following four types of the surveillance program services, which are provided by the US and European countries: cautionary information on patients with liver and renal disease, drug allergy cautionary information, clinical overuse and abuse, and gender-based cautionary information.

### Questionnaire administration

The developed questionnaire was made accessible online on the KIDS’s web-based survey system. We informed 167 organizations (158 academic societies under the Korean Academy of Medical Sciences and 9 physician- and pharmacist-related associations) of the survey webpage and solicited responses via phone calls and official letters from researchers from KIDS, a Korean public institute (May 22–27, 2014). All collaborating organizations sent e-mails, including a link to our survey system to their registered members or posted the link on their websites. After 3 weeks (June 10–13), we contacted the organizations with low response rates for further solicitation. Nonetheless, the response rate remained low, raising concerns regarding the lack of representativeness. Therefore, we conducted face-to-face interviews using printed questionnaires among participants at general academic congresses for hospital pharmacists and physicians. The survey was conducted from May 23 to August 11, 2014.

### Statistical analysis

Data collected through face-to-face interviews were manually entered into a database, which was subsequently merged with the database constructed from the web-based survey. Questions were structured in a manner to collect categorical variables and calculate the frequency and percentage (%). We calculated the frequency and percentage of responses according to the characteristics of study participants, such as profession, gender, age, region, years of practice, type of medical institution or pharmacy, and specialty (for physicians only). For the questions relating to the level of satisfaction with the medication surveillance program, whether the medication surveillance had resulted in specific improvements, and the degree to which each type of surveillance service improved clinical practice, we calculated the frequency by profession and performed a Pearson’s chi-square test. To determine whether there was a difference between the opinions of physicians and pharmacists regarding additional medication surveillance services needed, we conducted a two-sample Z test on the proportions of respondents who indicated each type of the additional medication surveillance service.

To identify the factors influencing the levels of satisfaction with the medication surveillance program, we conducted a multiple ordinal logistic regression analysis by adjusting for the characteristics of study participants (profession, gender, age, region, years of practice, type of medical institution or pharmacy, and specialty). To evaluate the representativeness of respondents, we performed a two-sample Z test on the proportions of physicians and pharmacists according to gender and region using demographic statistics of physicians and pharmacists. All statistical analyses were performed at the significance level of 0.05 using SAS 9.3 (SAS Institute Inc., Cary, NC, USA) [12, 13].

## Results

The study population was comprised of 93,094 physicians and 32,572 pharmacists, who were the registered members of 167 organizations (158 academic societies under the Korean Academy of Medical Sciences and 9 physician- and pharmacist-related associations). We collected completed questionnaires from 1164 participants: 515 physicians (response rate, 0.55%), 634 pharmacists (1.95%), and 15 of unknown profession. Among the 1164 healthcare professionals, 508 responded to paper-based interviews and 656 to the web-survey. After excluding 44 respondents (12 physicians, 17 pharmacists, and 15 no answer) who failed to answer most of the questions, we analyzed data from 1120 respondents.

Out of a total of 1120 respondents, 503 were physicians (44.9%), 617 pharmacists (55.1%), and 579 males (51.7%). The majority of the respondents were in their 30s (39.0%) and 40s (26.3%). The greatest proportion (26.6%) of the respondents had practiced their respective profession for less than 5 years, whereas 22.3% had practiced for 20 years or longer. The greatest proportion of physicians worked at tertiary hospitals (42.7%) and 41.2% of the pharmacists worked at hospital pharmacies. Most of the respondents (65.4%) were located in the metro areas of Seoul, Gyeonggi, and Incheon (Table 1). Seoul provided a disproportionately high proportion of respondents (Appendix 1).

**Table 1:** Characteristics of survey respondents: physicians and pharmacists in South Korea

Characteristics	Total no. of respondents	(%)	Physicians (%)		Pharmacists (%)		*p*-value*
Gender							<.0001
Male	579	(51.7)	366	(72.8)	213	(34.5)	
Female	541	(48.3)	137	(27.2)	404	(65.5)	
Age (years)							<.0001
20–29	106	(9.5)	25	(5.0)	81	(13.1)	
30–39	437	(39.0)	206	(41.0)	231	(37.4)	
40–49	294	(26.3)	128	(25.4)	166	(26.9)	
50–59	180	(16.1)	89	(17.7)	91	(14.7)	
60+	103	(9.2)	55	(10.9)	48	(7.8)	
Region							0.04
Seoul	475	(42.4)	223	(44.3)	252	(40.0)	
Gyeonggi-do	217	(19.4)	97	(19.3)	120	(19.4)	
Incheon	40	(3.6)	16	(3.2)	24	(3.9)	
Gangwon-do	29	(2.6)	12	(2.4)	17	(2.8)	
Daejeon	36	(3.2)	12	(2.4)	24	(3.9)	
Chungcheongbuk-do	20	(1.8)	16	(3.2)	4	(0.6)	
Chungcheongnam-do	28	(2.5)	17	(3.4)	11	(1.8)	
Gwangju	18	(1.6)	8	(1.6)	10	(1.6)	
Jeollabuk-do	31	(2.8)	15	(3.0)	16	(2.6)	
Jeollanam-do	21	(1.9)	8	(1.6)	13	(2.1)	
Daegu	52	(4.6)	18	(3.6)	34	(5.5)	
Ulsan	16	(1.4)	5	(1.0)	11	(1.8)	
Busan	65	(5.8)	26	(5.2)	39	(6.3)	
Gyeongsangbuk-do	26	(2.3)	11	(2.2)	15	(2.4)	
Gyeongsangnam-do	36	(3.2)	18	(3.6)	18	(2.9)	
Jeju	10	(0.9)	1	(0.2)	9	(1.5)	
Career (years)							0.68
0–4	298	(26.6)	126	(25.0)	172	(27.9)	
5–9	236	(21.1)	98	(19.5)	138	(22.4)	
10–14	193	(17.2)	92	(18.3)	101	(16.4)	
15–19	97	(8.7)	44	(8.7)	53	(8.6)	
20+	250	(22.3)	114	(22.7)	136	(22.0)	
Missing	46	(4.1)	29	(5.8)	17	(2.8)	
Hospital or Pharmacy type							<.0001
Tertiary hospital/ Hospital pharmacy	469	(41.9)	215	(42.7)	254	(41.2)	
Hospital/Pharmacy nearby hospital	228	(20.4)	68	(13.5)	160	(25.9)	
Clinic/Community pharmacy	366	(32.7)	201	(40.0)	165	(26.7)	
Missing	57	(5.0)	19	(3.8)	38	(6.2)	
Total	1120	(100.0)	503	(44.9)	617	(55.1)	

*p-value was calculated using chi-square test

Overall, 63.1% of all respondents were either strongly satisfied or satisfied with the medication surveillance program (19.2 and 43.9%, respectively). More pharmacists (69.1%) were satisfied than were physicians (55.6%). Among the respondents, 77.8% answered “agree” or “strongly agree” to the question regarding whether the surveillance program had decreased therapeutic duplication, and 63.8 and 63.3% of the respondents agreed that the surveillance program prevented ADRs and reduced drug overuse, respectively. Similar to the general satisfaction, more pharmacists were satisfied with the improvement conferred by the program than were physicians. The majority of members of both professions were satisfied with the decrease in therapeutic duplication (73.4% of physicians and 81.4% of pharmacists). However, physicians’ satisfaction (55.5%) was considerably lower regarding the prevention of ADRs than that of pharmacists (70.6%). The proportion of physicians satisfied with the decrease in drug overuse by physicians (61.9%) was similar to that of pharmacists (64.4%) (Table 2).

**Table 2:** Healthcare providers’ satisfaction with the medication surveillance program in South Korea

Statement	Frequency (%)					*p*-value^*^
	Strongly unsatisfied	Unsatisfied	Neutral	Satisfied	Strongly satisfied	
Are you satisfied with the medication surveillance?	27 (2.4)	61 (5.5)	323 (29.0)	488 (43.9)	213 (19.2)	< 0.001
Physicians	22 (4.4)	49 (9.8)	151 (30.2)	210 (42.0)	68 (13.6)	
Pharmacists	5 (0.8)	12 (2.0)	172 (28.1)	278 (45.4)	145 (23.7)	
Do you think the medication surveillance has improved the following?						
Prevention of adverse drug reactions	21 (1.9)	103 (9.3)	278 (25.0)	468 (42.1)	241 (21.7)	< 0.001
Physicians	19 (3.8)	70 (14.1)	133 (26.7)	197 (39.6)	79 (15.9)	
Pharmacists	2 (0.3)	33 (5.4)	145 (23.7)	271 (44.2)	162 (26.4)	
Decrease in therapeutic duplication	17 (1.5)	65 (5.8)	165 (14.8)	440 (39.5)	426 (38.3)	< 0.001
Physicians	13 (2.6)	34 (6.8)	86 (17.2)	204 (40.8)	163 (32.6)	
Pharmacists	4 (0.7)	31 (5.1)	79 (12.9)	236 (38.5)	263 (42.9)	
Decrease in drug overuse	27 (2.4)	112 (10.1)	269 (24.2)	432 (38.8)	272 (24.5)	< 0.001
Physicians	23 (4.6)	52 (10.4)	115 (23.0)	197 (39.5)	112 (22.4)	
Pharmacists	4 (0.7)	60 (9.8)	154 (25.1)	235 (38.3)	160 (26.1)	

*p-value was calculated using chi-square test

Pharmacists were more than two-times more likely to be satisfied with the medication surveillance program than were physicians [adjusted odds ratio (AOR), 2.13; 95% confidence interval (CI), 1.65–2.76]. Respondents aged 60 years and older (AOR, 2.63; 95% CI, 1.36–5.09) were more likely to be satisfied than were those in their 20s. Gender, region, and years of practice did not influence satisfaction levels (Table 3). Similarly, the clinical specialty of the physicians did not influence the satisfaction levels (Appendix 2).

**Table 3:** Logistic regression model of factors associated with healthcare professionals’ satisfaction with the medication surveillance program

Independent variables	OR (95% CI) (modeling the likelihood of being classified into a higher level of satisfaction)	Adjusted OR (95% CI)^a^ (modeling the likelihood of being classified into a higher level of satisfaction)
Profession		
Physician	1.00	1.00
Pharmacists	2.02 (1.62–2.52)	2.13 (1.65–2.76)
Gender		
Male	1.00	1.00
Female	1.07 (0.87–1.33)	1.00 (0.77–1.31)
Age (years)		
20–29	1.00	1.00
30–39	0.94 (0.64–1.39)	1.18 (0.75–1.87)
40–49	1.03 (0.68–1.55)	1.04 (0.61–1.76)
50–59	1.63 (1.05–2.55)	1.72 (0.93–3.17)
60+	2.20 (1.33–3.64)	2.63 (1.36–5.09)
Region		
Metropolitan	1.00	1.00
City	1.12 (0.83–1.50)	0.99 (0.72–1.36)
Rural	1.16 (0.87–1.54)	1.07 (0.78–1.47)
Career (years)		
< 5	1.00	1.00
5–9	0.83 (0.61–1.14)	0.80 (0.56–1.13)
10–14	1.11 (0.80–1.55)	1.07 (0.73–1.57)
15–19	1.13 (0.74–1.73)	1.05 (0.64–1.74)
20+	1.56 (1.14–2.12)	1.06 (0.68–1.66)
Hospital or Pharmacy type		
Tertiary hospital/Hospital pharmacy	1.00	1.00
Hospital/Pharmacy nearby hospital	1.85 (1.38–2.49)	1.62 (1.18–2.21)
Clinic/Community pharmacy	1.33 (1.03–1.71)	1.22 (0.92–1.61)

*AOR* Adjusted odds ratio, *IC* Confidence interval

^a^Adjusted for profession, gender, age, region, years of practice, type of medical institution or pharmacy, and specialty

Among the four types of medication surveillance services, the drug–drug interaction information was viewed as the most useful; 26.9 and 55.7% answered “agree” or “strongly agree,” respectively, to the question regarding its usefulness, and 78.3% of physicians and 86.1% of pharmacists viewed the drug–drug interaction information as useful. Overall, 74.3, 72.5, and 69.3% viewed pregnancy contraindication, age contraindication, and therapeutic duplication, respectively, as useful; more pharmacists regarded these types of information to be more useful than did physicians (Table 4).

**Table 4:** Usefulness of the medication surveillance program in a clinical setting per total respondents: physicians and pharmacists

Medication surveillance program	Frequency (%)					*p*-value
	Very un-useful	Somewhat un-useful	Neutral	Somewhat useful	Very useful	
Drug–drug interaction	11 (1.0)	49 (4.4)	133 (12.0)	299 (26.9)	618 (55.7)	< 0.001
Physicians	10 (2.0)	34 (6.8)	64 (12.9)	161 (32.4)	228 (45.9)	
Pharmacists	1 (0.2)	15 (2.4)	69 (11.3)	138 (22.5)	390 (63.6)	
Age contraindication: Pediatric, Elderly	20 (1.8)	59 (5.3)	226 (20.4)	361 (32.5)	444 (40.0)	< 0.001
Physicians	17 (3.4)	39 (7.8)	129 (26.0)	166 (33.4)	146 (29.4)	
Pharmacists	3 (0.5)	20 (3.3)	97 (15.8)	195 (31.8)	298 (48.6)	
Pregnancy contraindication	28 (2.5)	58 (5.2)	198 (17.9)	295 (26.7)	526 (47.6)	< 0.001
Physicians	24 (4.9)	38 (7.7)	101 (20.4)	143 (28.9)	188 (38.1)	
Pharmacists	4 (0.7)	20 (3.3)	97 (15.9)	152 (24.9)	338 (55.3)	
Therapeutic duplication	18 (1.6)	76 (6.9)	245 (22.2)	361 (32.6)	406 (36.7)	< 0.001
Physicians	17 (3.4)	45 (9.1)	131 (26.5)	172 (34.8)	129 (26.1)	
Pharmacists	1 (0.2)	31 (5.1)	114 (18.6)	189 (30.9)	277 (45.3)	

* *p*-value was calculated using chi-square test

Cautionary information for patients with liver or renal diseases was identified as the medication surveillance service of greatest need; it was selected by 48.7% of all respondents (55.0% of physicians, 43.7% of pharmacists), followed by drug-allergy caution information (33.8%), drug abuse caution information (13.6%), and gender caution information (3.9%). When stratified by type of healthcare provider, the proportion of frequency was significantly different between physicians and pharmacists regarding safety information on liver or renal diseases and drug interactions (55.0% vs. 43.7%) and clinical abuse or misuse (10.6% vs. 16.0%) (Table 5).

**Table 5:** Additional safety information most needed per total respondents: physicians and pharmacists

Safety information	Frequency (%)						*p*-value^*^
	Total		Physicians		Pharmacists		
Liver or kidney disease–drug interaction	512	(48.7)	255	(55.0)	257	(43.7)	< 0.001
Drug allergy	356	(33.8)	147	(31.7)	209	(35.5)	0.02
Clinical abuse, misuse	143	(13.6)	49	(10.6)	94	(16.0)	< 0.001
Precautions due to gender	41	(3.9)	13	(2.8)	28	(4.8)	0.21
Total	1052	100	464	100	588	100	

* *p*-value was calculated using two-sample Z test

## Discussion

### Difference in satisfaction between professions

Overall, 63.0% of the survey respondents were “satisfied” or “very satisfied” with the medication surveillance program, and a decrease in therapeutic duplication was regarded as a benefit of the medication surveillance by most respondents. However, the odds of being satisfied with the medication surveillance program were two-times greater among pharmacists than among physicians, which suggests the need to improve factors hindering satisfaction with the medication surveillance program by considering the difference in the clinical status between the professions. While profession influenced the level of satisfaction with the surveillance program, the gender, region, and years of practice did not. Furthermore, profession influenced how the respondents felt about the improvements and clinical usefulness of the surveillance program. This finding may be attributable to differences in clinical characteristics, such as practice setting, the main types of service for which the medication surveillance program was used, and the types of relationships between physicians and pharmacists and their respective patients.

First, the observed differences between physicians and pharmacists may be caused by the fact that prescribing and dispensing occurred sequentially. In Korea, the medication surveillance program is provided via pop-up alerts through real-time checks of prescriptions at the stage of prescribing and dispensing. The physicians first access the surveillance program alerts when they prescribe medication, and the pharmacists access the alerts that the physicians leave unchecked. For that reason, the physicians and the pharmacists may feel differently about the amount and content of the alerts.

In addition, satisfaction level can be influenced by the fact that physicians and pharmacists focus on different areas in the process of prescribing and dispensing. Pharmacists focus on pharmacological treatment but prescribing by physicians is influenced by various factors, such as uncertainty associated with diagnosis, perception of patients’ expectations, and need to maintain good physician–patient relationships [14, 15]. In the areas that need mutual collaboration, pharmacists want to identify and manage patients’ drug-related problems, but physicians want to improve patients’ adherence [16]. Because of the differences in the scope of roles and areas of focus, physicians and pharmacists might differently value the importance of alerts that provide information on drugs only, leading to the difference in levels of satisfaction.

Furthermore, different professionals may feel differently about medication surveillance alerts. The results showed that 73.4% of physicians and 81.4% of pharmacists (8.0% difference) answered “agree” or “strongly agree” to the question regarding whether therapeutic duplication decreased through medication surveillance. This finding suggests that both physicians and pharmacists positively perceived the effect of the surveillance program on the reduction of therapeutic duplication because medications prescribed at another institution and dispensed at another pharmacy could frequently change. Nonetheless, a 15.1% difference was noted among professions who agreed that ADR prevention was a benefit of medication surveillance. Compared to pharmacists, highly specialized physicians deal with a limited scope of medications, and therefore, are more likely to receive alerts for which they already know the details and for which they perceive little value. This finding may have increased the inconvenience healthcare providers felt about the medication surveillance program, and thus, decreased their satisfaction with the surveillance regarding ADR prevention.

### Factors affecting satisfaction

Our respondents, especially physicians, found age and pregnancy contraindication information to be less useful than the drug–drug interaction. Age and pregnancy contraindications are primarily based on the chemical structure of drugs and the results of animal studies because there are few clinical data. As previously mentioned, physicians consider satisfying patients’ expectations regarding treatment, building trusting relationships, and clinical relevance of medicine. This suggests that they may have low levels of utilization and satisfaction with alerts that provide information based on pharmacological theories when prescribing for children and pregnant women.

Moreover, both physicians and pharmacists cited alert fatigue as the most important reason for their dissatisfaction with alerts. Alert fatigue may negatively influence the level of satisfaction with medication surveillance. Healthcare professionals receive the same age and pregnancy contraindication alerts as the patient’s age or pregnancy status remains unchanged. In particular, physicians, who tend to see the same patient for a longer period of time than do pharmacists, may receive the same alerts over a longer period. This situation can be seen in the difference between the percentage of pharmacists and physicians who viewed age and pregnancy contraindication information as being clinically more useful (17.6 and 13.2%, respectively) than the drug–drug interaction information (7.8%).

To eliminate the factors negatively influencing satisfaction with the medication surveillance program, efforts to provide only clinically significant and highly effective surveillance services are needed through a revaluation of the effects on drug use and value of currently provided information. The US and European countries that have a longer history of implementing medication surveillance have experienced the alert fatigue phenomenon [8, 9]. This fatigue may not only hinder tasks of physicians and pharmacists but may also lead them to be unaware of truly important alerts that need their attention. In an effort to solve the problem, the Netherlands strengthened the criteria for developing a drug database for the medication surveillance program by considering the effect [11, 17]. This criterion was to provide only clinically meaningful and effective information that could prevent inappropriate drug use that was likely to recur through evaluating the potential effect of the surveillance program on behavioral change in prescribing and dispensing. Based on the literature review, drug–drug interaction was classified into five levels from no evidence to controlled, published interaction studies in patients or healthy volunteers, and clinically relevant endpoints according to the quality of scientific evidence. According to the severity level of specific clinical effects or toxicities of interactions, drug–drug interactions were classified on a 6-category scale from clinically irrelevant effects to multi-organ failure/death. Following the classifications, a group of experts decided whether there were drug–drug interactions and a need for intervention through the electronic medical surveillance system. Experts selected only the information that had drug–drug interaction and required action. In addition, the Netherlands has improved the medication surveillance system in which the prescriber can control the repetition of alerts and revaluate the appropriateness of the existing drug database in the surveillance program.

### Current positive effect of the program and future needs

Gender, region, and years of practice did not influence the level of satisfaction with medication surveillance, because the surveillance program in Korea was developed and disseminated under the leadership of the government and most healthcare professionals use the same software through the nationwide computerized medication surveillance system. Nonetheless, the respondents over 60 years of age were significantly associated with a higher level of satisfaction with the program (AOR, 2.63; 95% CI, 1.36–5.09). This result is interesting considering the possibility that exposure to alerts for a long period of time could cause alert fatigue among healthcare professionals. However, there may be little difference in the duration of exposure between age groups in this study, in part because at the time of the survey, a long enough time has not passed since the implementation of the surveillance system. The higher level of satisfaction among older respondents may be attributed to the educational effect of the warning messages; recent pharmacological issues may be new to older physicians but not to recent medical graduates.

Another survey conducted among physicians and pharmacists before the establishment of the computerized medication surveillance system indicated that some of the healthcare professionals prescribed or dispensed contraindicated drugs because they did not know about the contraindication, emphasizing the need for a system that continuously checks drug information [18]. The current medication surveillance system was shown to reduce drug–drug interactions [19]. Consistent with findings of positive effects of medication surveillance program in other countries [20, 21], our survey also showed that healthcare professionals cited reductions in therapeutic duplication, ADR, and drug overuse as the benefits of the medication surveillance system. These findings suggest the strengths of Korea’s medication surveillance system that guarantees equitable access to the program services for most healthcare providers nationwide.

Despite the strengths, the medication surveillance system needs to be customized to meet the needs of individual patients to provide various kinds of information that are more helpful for patient safety and relevant in clinical practice. We identified the areas of high demand, such as cautionary information regarding patients with liver and renal diseases (43.7%) and drug-allergy cautionary information (35.5%). This type of information can be made available only if the surveillance system considers clinical information of individual patients, such as diagnosis, laboratory results, patient history, and medication history. Both the US and European countries provide a wider scope in the medication surveillance program that includes drug-allergy cautions, drug-disease cautions, and abuse/overuse cautions [8, 9]. Cautionary information for patients with renal disease was identified as the most useful of all types of medication surveillance services in the Netherlands where cautionary information is customized based on the renal function test of patients [22, 23]. To provide such diverse safety information, it is necessary to develop a system that collects patients’ lab results, with their consent, and automatically checks their clinical information at the time of prescribing and dispensing. Implementation of this system will require an electronic medical record system, integration with the prescribing and dispensing system, and the patients’ understanding and active participation in the medication surveillance program.

In our survey, some respondents answered the open-ended question and expressed their desire to retrieve additional information other than the items that we suggested, such as the list of drugs that the patients were currently taking, diagnoses, and medication history. This desire suggests that the current system of providing surveillance alerts on contraindications itself is not enough to be useful in clinical practice. Detailed information on contraindicated drugs was reported to be in high demand in other countries; information on side effects and the pharmacological mechanism are provided in the US and European countries [24]. Moreover, other countries have customized their medication surveillance systems to meet the needs of individual patients and established a system to share patient information among various healthcare providers; the US introduced E-prescribing systems and established the Web Portal Rx Hub by which healthcare providers can retrieve both patient history and prescription details. Since 2008, the Netherlands has a system that exchanges patient information [8, 22]. Korea also needs to establish a more effective and advanced medication surveillance system that is customizable to the needs of individual patients and healthcare professionals.

### Strengths and limitations

This study diagnosed the status of the medication surveillance program, which has evolved over the past decade in Korea, by examining the level of satisfaction among healthcare providers with the current surveillance program and identified areas of additional needs for further development of the medication surveillance system. Despite its significance, this study had its limitations. Our sample may not represent the overall healthcare provider population. The respondents of our study were mainly in their 30s and 40s. This may be because younger respondents were more likely to be familiar with a web-based survey and sensitive to changes related to their work. The high representation of younger respondents could underestimate the overall level of satisfaction with the medication surveillance program, because respondents over 60 years of age were associated with higher levels of satisfaction in the logistic regression analysis. To mitigate the age-related selection bias, we additionally conducted face-to-face interviews to recruit older physicians; this attempt may contribute to the higher level of satisfaction with the program. Additionally, a substantial proportion of the respondents work in tertiary hospitals (42.7%) and hospital pharmacies (41.2%). These highly trained physicians and pharmacists may have been more interested in the medication surveillance program implemented by the government and, thus, participated in the survey more actively than did their peers in other settings. However, their special interest in the program or medicines does not guarantee their preferences or satisfaction with the program. The higher proportion of these respondents may not have affected the level of satisfaction. Rather, the lack of respondents working in hospitals or pharmacies near hospitals lead to underestimation of the level of satisfaction. These characteristics of our sample may hinder the generalizability of our findings. Future research should be conducted with respondents who are representative of the healthcare provider population in Korea, and thus, enhance the validity of the results of the study. In addition, as our study showed only clinical needs, future research needs to investigate factors influencing satisfaction.

## Conclusions

The results of this study suggest that the current medication surveillance system should be improved by providing appropriate information that can be helpful depending on the interests and roles of physicians and pharmacists, as well as satisfying their clinical needs for additional information. Further development of the medication surveillance program should focus on clinically significant and useful information in practice through evaluating the effect of potential information on the behavioral change of healthcare providers rather than on a mere increase in information quantity. In addition, besides the clinical needs of physicians and pharmacists, future research needs to determine factors influencing satisfaction by profession.

## Data Availability

The data used and/or analyzed in the study were used under agreement for the current study and are not publicly available.

## Appendix 1

**Table 6:** Characteristics of survey respondents regarding the medication surveillance program

Characteristics	Physicians					Pharmacists				
	Total national population (%)		No. of respondents (%)		*p*-value^*^	Total national population (%)		No. of respondents (%)		*p*-value^*^
Gender										
Male	81,000	(81.0)	366	(72.8)	< 0.01	14,000	(38.9)	213	(34.5)	0.04
Female	19,000	(19.0)	137	(27.2)	0.02	22,000	(61.1)	404	(65.5)	0.04
Region										
Seoul	27,490	(29.5)	223	(44.3)	< 0.01	8215	(25.4)	252	(40.0)	< 0.01
Gyeonggi-do	17,564	(19.0)	97	(19.3)	0.80	6966	(21.3)	120	(19.4)	0.24
Incheon	4170	(4.5)	16	(3.2)	0.17	1503	(4.6)	24	(3.9)	0.10
Gangwon-do	2492	(2.7)	12	(2.4)	0.70	893	(2.7)	17	(2.8)	0.92
Daejeon	3316	(3.6)	12	(2.4)	0.16	1083	(3.3)	24	(3.9)	0.13
Chungcheongbuk-do	2249	(2.4)	16	(3.2)	0.24	939	(3.0)	4	(0.6)	< 0.01
Chungcheongnam-do	2859	(3.1)	17	(3.4)	0.67	1161	(3.6)	11	(1.8)	0.02
Gwangju	3176	(3.4)	8	(1.6)	0.03	1082	(3.3)	10	(1.6)	0.02
Jeollabuk-do	3369	(3.6)	15	(3.0)	0.46	1270	(3.9)	16	(2.6)	0.10
Jeollanam-do	2898	(3.1)	8	(1.6)	0.05	1156	(3.5)	13	(2.1)	0.05
Daegu	5247	(5.6)	18	(3.6)	0.05	7161	(5.5)	34	(5.5)	0.96
Ulsan	1545	(1.7)	5	(1.0)	0.25	601	(1.8)	11	(1.8)	0.94
Busan	7386	(8.0)	26	(5.2)	0.02	2370	(7.3)	39	(6.3)	0.33
Gyeongsangbuk-do	3475	(3.7)	11	(2.2)	0.07	1392	(4.3)	15	(2.4)	0.02
Gyeongsangnam-do	4778	(5.1)	18	(3.6)	0.12	1740	(5.4)	18	(2.9)	< 0.01
Jeju	961	(1.0)	1	(0.2)	0.06	363	(1.1)	9	(1.5)	0.36

* *p*-value was calculated with the two-sample Z test

## Appendix 2

**Table 7:** Logistic regression model of factors associated with healthcare professionals’ satisfaction with the medication surveillance program

Characteristics	Total no. of respondents^a^	(%)	OR (95% CI) (modeling the likelihood of being classified into a higher level of satisfaction)
Specialty			
Family medicine	53	(10.5)	1.00
Internal medicine	72	(14.3)	1.16 (0.60–0.22)
Anesthesiology	23	(4.6)	0.69 (0.28–1.70)
Radiation oncology	2	(0.4)	0.37 (0.03–4.83)
Urology	21	(4.2)	1.41 (0.55–3.59)
Obstetrics & Gynecology	41	(8.2)	0.70 (0.33–1.48)
Plastic surgery	1	(0.2)	34.62 (0.53 to > 999.99)
Pediatrics & Adolescent medicine	29	(5.8)	0.80 (0.35–1.84)
Neurology	27	(5.4)	0.27 (0.12–0.63)
Neurosurgery	9	(1.8)	0.79 (0.22–2.89)
Ophthalmology	4	(0.8)	4.42 (0.79–24.67)
Radiology	11	(2.2)	2.95 (0.88–9.94)
Preventive Medicine	1	(0.2)	2.33 (0.06–92.87)
General surgery	18	(3.6)	1.15 (0.13–3.08)
Emergency medicine	7	(1.4)	0.78 (0.18–3.28)
Otorhinolaryngology	25	(5.0)	0.45 (0.19–1.09)
Rehabilitation medicine	15	(3.0)	1.09 (0.38–3.12)
Neuropsychiatry	14	(2.8)	1.22 (0.41–3.62)
Dentistry	22	(4.4)	2.09 (0.83–5.27)
Dermatology	39	(7.8)	1.02 (0.48–2.18)
Thoracic & Cardiovascular surgery	5	(1.0)	0.82 (0.15–4.38)
Orthopedics	12	(2.4)	0.67 (0.21–2.11)
Others	16	(3.2)	2.21 (0.80–6.11)
Missing	36	(7.2)	
Total	503	(100)	

^a^Physicians may have two or more specialties

## Abbreviations

ADRs: Adverse Drug Reactions
AOR: Adjusted Odds Ratio
CI: Confidence Interval
KIDS: Korea Institute of Drug Safety and Risk Management
MFDS: Ministry of Food and Drug Safety
